# Hedgehog Signaling Overcomes an EZH2-Dependent Epigenetic Barrier to Promote Cholangiocyte Expansion

**DOI:** 10.1371/journal.pone.0168266

**Published:** 2016-12-09

**Authors:** Nidhi Jalan-Sakrikar, Thiago M. De Assuncao, Jie Lu, Luciana L. Almada, Gwen Lomberk, Martin E. Fernandez-Zapico, Raul Urrutia, Robert C. Huebert

**Affiliations:** 1 Division of Gastroenterology and Hepatology, Mayo Clinic and Foundation, Rochester, MN, United States of America; 2 Gastroenterology Research Unit, Mayo Clinic and Foundation, Rochester, MN, United States of America; 3 Schultze Center for Novel Therapeutics, Mayo Clinic and Foundation, Rochester, MN, United States of America; 4 Center for Cell Signaling in Gastroenterology, Mayo Clinic and Foundation, Rochester, MN, United States of America; University of Navarra, SPAIN

## Abstract

**Background & Aims:**

Developmental morphogens play an important role in coordinating the ductular reaction and portal fibrosis occurring in the setting of cholangiopathies. However, little is known about how membrane signaling events in ductular reactive cells (DRCs) are transduced into nuclear transcriptional changes to drive cholangiocyte maturation and matrix deposition. Therefore, the aim of this study was to investigate potential mechanistic links between cell signaling events and epigenetic regulators in DRCs.

**Methods:**

Using directed differentiation of induced pluripotent stem cells (iPSC), isolated DRCs, and *in vivo* models, we examine the mechanisms whereby sonic hedgehog (Shh) overcomes an epigenetic barrier in biliary precursors and promotes both cholangiocyte maturation and deposition of fibronectin (FN).

**Results:**

We demonstrate, for the first time, that Gli1 influences the differentiation state and fibrogenic capacity of iPSC-derived hepatic progenitors and isolated DRCs. We outline a novel pathway wherein Shh-mediated Gli1 binding in key cholangiocyte gene promoters overcomes an epigenetic barrier conferred by the polycomb protein, enhancer of zeste homolog 2 (EZH2) and initiates the transcriptional program of cholangiocyte maturation. We also define previously unknown functional Gli1 binding sites in the promoters of cytokeratin (CK)7, CK19, and FN. Our *in vivo* results show that EZH2 KO mice fed the choline-deficient, ethanolamine supplemented (CDE) diet have an exaggerated cholangiocyte expansion associated with more robust ductular reaction and increased peri-portal fibrosis.

**Conclusion:**

We conclude that Shh/Gli1 signaling plays an integral role in cholangiocyte maturation *in vitro* by overcoming an EZH2-dependent epigenetic barrier and this mechanism also promotes biliary expansion *in vivo*.

## Introduction

Chronic biliary diseases and their complications continue to be the cause of significant morbidity and mortality and these disorders have very limited therapeutic options[1]. Cholangiocytes, the cells lining the biliary tree, are targets of a heterogeneous group of end-stage liver diseases, known as the cholangiopathies, that are currently untreatable without liver transplantation [2]. These disorders are associated with a robust ductular reaction, typified by biliary expansion and extracellular matrix (ECM) deposition that ultimately lead to progressive peri-portal fibrosis [3]. Diseased cholangiocytes are thought to initiate this maladaptive repair process via several mechanisms, including their influence on the maturation state of biliary precursors. DRCs are small, immature, ductular cells that are scarcely detectable in the normal liver, but highly expanded during the cholangiopathies, tightly associated with ECM molecules, and thought to play a role in the coordination of biliary repair [4]. While the origins and cellular fate of DRC continue to be defined, it remains likely that a portion of this diverse population of cells undergoes maturation toward mature cholangiocytes in the setting of biliary damage. The pathobiologic mechanisms regulating the maturation of DRCs toward cholangiocytes and how these processes may contribute to the progression of fibrosis are understudied areas and remain poorly understood. Thus, it is necessary to better elucidate the mechanisms regulating biliary differentiation in order to identify fibrogenic pathways that can be targeted therapeutically.

iPSC are derived from somatic cells by forced expression of various pluripotency factors, rendering them capable of self-renewal and diverse differentiation [5]. iPSC-derived hepatocyte-like cells have been extensively utilized to model hepatocellular disorders [6, 7], test new pharmacologic compounds [8], and for regenerative medicine applications [9]. Based on improving understanding of liver development[10], our group and others have recently extended this technology by developing unique approaches for the directed differentiation of cholangiocytes from iPSC [11–14]. While this emerging technology will clearly be utilized for modeling the cholangiopathies, to develop biliary pharmacology, and in cell therapy applications, we demonstrate here that it is also useful as an *in vitro* model of the stem cell-to-cholangiocyte transition in general and provides basic insights into biliary maturation and the ductular reaction that occurs in biliary disease.

Shh is a developmental morphogen with classical roles in embryologic patterning and morphogenesis in a variety of species and tissues. More recently, the Shh pathway has been implicated in regeneration and differentiation of stem cells [15] including fetal liver progenitor cells, where it was found to inhibit hepatocyte differentiation [16]. In the context of chronic liver disease, Shh and its downstream transcription factor, Gli2, have been extensively implicated in a variety of processes including regeneration [17], epithelial-mesenchymal transitions [18, 19], remodeling / repair after biliary obstruction [20–22] and cholangiocarcinoma [23]. Our study further corroborates Shh as a coordinator of the ductular reaction, but also implicates Gli1 and suggests a new epigenetic mechanism whereby activation of Shh and Gli1 in DRCs directly contributes to cholangiocyte maturation and expansion of the biliary compartment. The work is also in line with several studies suggesting that Shh signaling and progenitor cell activation may contribute to biliary fibrosis[24, 25].

Epigenetic modifications are increasingly implicated in liver cell differentiation in various liver diseases [26, 27]. The polycomb repressive complex 2 (PRC2) protein, EZH2, enzymatically mediates the tri-methylation of lysine 27 on histone 3 (H3K27me3) to inactivate gene expression [28]. EZH2 has been implicated in hepatoblast proliferation and differentiation in the embryonic liver [29]. Furthermore, epigenetic events are increasingly implicated in the desmoplastic ECM of the tumor microenvironment [30]. In aggregate, this raises the possibility of a common epigenetic regulatory program, mediated by EZH2 that not only drives biliary maturation, but also contributes to matrix deposition, processes that may therefore be inextricably linked and mutually-dependent.

In this study, we tested the overarching hypothesis that Shh interacts with immature biliary precursors, promoting cholangiocyte maturation and FN deposition, through EZH2-mediated mechanisms. We employed specific pharmacologic and genetic interventions in two complementary models of differentiation, iPSC and isolated DRC, along with the CDE diet in genetically-modified mice. This study reports the following novel findings: 1) Shh simultaneously promotes both cholangiocytic maturation in biliary precursors and release of FN; 2) Previously unknown regulatory sites within the promoters of CK7, CK19, and FN bind Gli1 upon Shh stimulation; 3) This process overcomes an epigenetic barrier and promotes cholangiocyte gene expression by reducing EZH2-dependent transcriptional repression; and 4) Animals genetically lacking EZH2 have an exaggerated response to the CDE diet with enhanced ductular reaction, including biliary proliferation, FN production, and peri-portal fibrosis. Overall, these findings identify new molecular targets involved in biliary regeneration and the associated fibrosis. Therefore, these studies not only provide new mechanistic insights, but also have important biomedical relevance.

## Materials and Methods

### Cholangiocyte culture

NHC cells were a gift from N.F. LaRusso. NHC and iPSC-derived cholangiocytes (iDC) were cultured in H69 media (DMEM/F12 supplemented with 10% fetal bovine serum, 1% penicillin/streptomycin, adenine, insulin, epinephrine, T3-T, hydrocortisone and epidermal growth factor) [12].

### Reprogramming / iPSC culture

Human skin fibroblasts were cultured from biopsy specimens with the approval of the Mayo Clinic Institutional Review Board—Subcommittee C (approval #14–001464). Participants provided written informed consent. These cells were induced to pluripotency by transient forced expression of OCT4, SOX2, KLF4, and c-MYC, using the Sendai system, as previously described [12]. iPSC were seeded in culture plates pre-coated with Geltrex (Life Technologies) and cultured in mTeSR culture medium (STEMCELL Technologies) with 1% penicillin/streptomycin.

### Generation of induced pluripotent stem cell-derived cholangiocytes (iDC)

**Fig 1:**
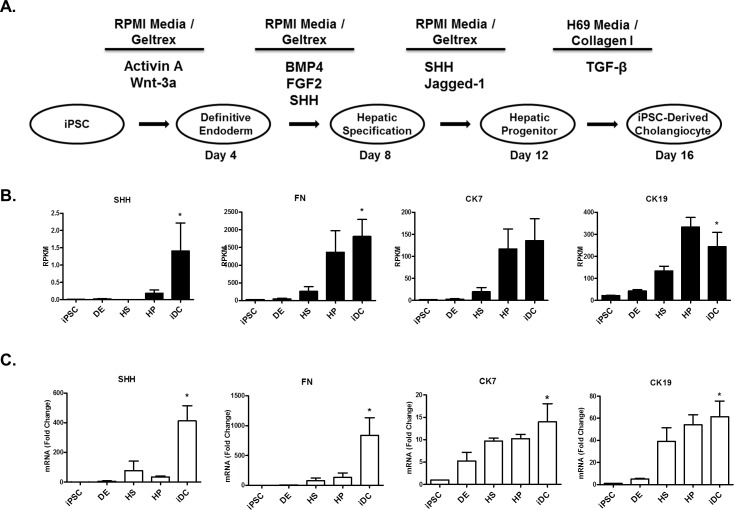
Shh activation and FN production accompany cholangiocyte differentiation. A. Stepwise differentiation toward cholangiocytes in five phases: induced pluripotent stem cells (iPSC), definitive endoderm (DE), hepatic specification (HS), hepatic progenitor (HP), and iPSC-derived cholangiocytes (iDC). B. RNA sequencing data showing upregulation of Shh, FN, and the biliary cytokeratins, CK7 and CK19. C. RT-PCR showing increases in Shh, FN, CK7, and CK19 mRNA levels. The data are shown as means ± standard error. *p < 0.05.

The step-wise differentiation toward iDCs was accomplished by a process of temporal exposure to biliary morphogens (Fig 1A), as previously described [12]. Differentiation proceeds in five phases: iPSC, definitive endoderm (DE), hepatic specification (HS), hepatic progenitor (HP), and iDC.

### shRNA and Inhibitor Treatment

EZH2 overexpression was accomplished with an adenovirus (EZH2Ad). A lentiviral shRNA construct against the Smoothened receptor (SMO-R) was used for knockdown of SMO-R. HEK293 cells were used to generate the lentivirus, which was then added to the differentiating cells at day1 of every phase beginning at the HS phase of differentiation. Cyclopamine (CPN) was obtained from Toronto Research Chemicals (Cat no. C988400) and dissolved in DMSO at concentration of 5 mM. DMSO (vehicle) or CPN was added to the cells at a final concentration of 5μM everyday during the differentiation starting at the HS phase.

### RNA Sequencing

RNA sequencing and bioinformatics analysis was conducted in collaboration with the Mayo Medical Genomics Facility as previously described [12]. Quality of the RNA was assessed by the Mayo Gene Expression Core using Agilent Bioanalyzers. All samples had RNA Integrity Numbers greater than 7.0. RNA sequencing was performed as paired-end base reads on an Illumina HiSeq 2000 with three samples per lane, using the TruSeq SBS Sequencing Kit, Version 3. Base calling was performed using Illumina’s RTA version 1.12.4.2. Bioinformatics were performed with the assistance of the Mayo Division of Biostatistics and Informatics. Analysis of each sample (alignment statistics, in-depth quality control metrics, and gene and exon expression levels) was done using Mayo Clinic’s MAPRSeq v1.2. Reads were mapped using Tophat version 2.0.6 against the hg19 reference genome and gene counts were produced using htseq. Differential expression analyses between samples were computed using an edgeR version 3.3.8 algorithm. Data was deposited in the NCBI Gene Expression Omnibus (accession number GSE86007).

### Ductular Reactive Cell Isolation

While several protocols exist for the isolation of rodent DRCs [31, 32], we chose to use a commonly used protocol for isolation of mouse DRCs [33, 34] utilizing immunomagnetic bead separation to enrich for cells containing epithelial cell adhesion molecule (EPCAM), a well-characterized progenitor cell marker [35].

### Western Blotting

Protein isolation was done with RIPA buffer. Nuclear extraction was performed using a commercially available kit (Abcam). Concentration was determined using a Spectrophotometer. 25 μg of protein was loaded onto 4–20% Tris-Glycine gels, electrophoresed, and transferred onto nitrocellulose membranes (Scientific Laboratory Supplies) for blotting. The membrane was blocked, incubated with primary antibodies (S1 Table), rinsed with TBST, and incubated with appropriate secondary antibodies. After washing, the membrane was exposed to luminol (Santa Cruz Biotechnology) and developed.

### Quantitative RT-PCR

Total RNA was extracted from cells using the RNeasy Plus Mini Kit (Qiagen). Reverse transcription was performed with 5 μg RNA using oligo (dT) primer and SuperScript III. Real-time PCR was performed in a volume of 25 μl using Sybr Green Master Mix and the 7500 Real-Time PCR System (Applied Biosystems). Primer sequences are shown in S2 Table.

### Immunohistochemistry (IHC)

Whole liver was harvested, sliced, formalin-fixed, and embedded in paraffin. Sections were cut to 4–8 μm and antigen unmasked with citrate. After quenching of endogenous peroxidase, the sections were blocked, and incubated with antibody against EZH2, FN, or CK7 (S1 Table) overnight @ 4°C. The remaining steps were carried out using an immunoperoxidase detection Kit (Vector Laboratories). Hematoxylin/Eosin, Masson’s trichrome stain and pico Sirius red stains were performed by the Mayo Histology Core. Quantification of 10 random portal tracts per group was performed using Image J software.

### Luciferase Assays

A 1.7-kb region upstream from the first exon of FN gene, a 2.5-kb region upstream the first exon of CK19 gene, and 1.1-kb region upstream the first exon of CK7 were amplified from human genomic DNA using PCR. The primers used for amplification of FN and CK19 promoter were designed with NheI and HindIII sites (FN—forward, 5’-GCTAGCATTCAGTTCGGGTC-3’; and reverse, 5’-AAGCTTCTCTCAGTAAAGCGC-3’/ CK19 –forward, 5’- TTTGCTAGCCAGCTGGATCACGGAG-3’; and reverse– 5’-ATAAAGCTTCTAGTTAGGGCGGCTGGTG-3’). The amplified products were transferred into NheI and HindIII site of the pGL3 basic vector (Promega). The CK7 promoter was purchased from Active Motif (S707713). The promoter region was digested with Mlu1 and Bgl2 enzymes and transferred into the Mlu1 and Bgl2 sites of the pGL3 basic vector. These constructs were named: “full FN promoter” or “−1693/+160 FN”; “full CK19 promoter” or “-2306/+203 CK19”; and “full CK7 promoter” or “-1002/+32 CK7” (+1 denotes the transcription initiation point of exon 1). *In silico* analyses of the FN promoter indicated 5 Gli1 binding sites. Deletion construct was designed using restriction enzymes in order to remove the first Gli1 binding site. The CK7, CK19 and FN full promoters were digested with BstXI+BglI, NcoI+BglI and NheI+BglI, respectively The fragments were filled in using a Klenow fragment and Pfu DNA polymerase and then ligated with T4 DNA ligation giving origin to a 357-bp CK7 truncation promoter, 1832-bp CK19 truncation promoter and 1018-bp truncation FN promoter. All constructs were confirmed by sequencing. HEK 293 cells (1 × 10^6^) were transfected using Effectene transfection reagent (Qiagen) with 0.5 μg of reporter plasmid containing various lengths of the 5′ promoter region of the human FN gene or 5′ promoter region of the human CK19 or CK7 gene as indicated, and, in some experiments, the cells were infected an adenoviral construct to overexpress EZH2 or treated with 5uM of EZH2 inhibitor GSK126 (ApexBio). After 48 h of transfection, some cells were stimulated for 18 h with Shh (100 ng/ml) and lysed, and relative luciferase activity was measured using the luciferase assay system (Promega) and a Turner 20/20 luminometer.

### Chromatin Immunoprecipitation (ChIP)

ChIP assays were performed at DE, HS, and HP phases of our iPSC-to-cholangiocyte differentiation protocol. At the last day of each phase, the cells were fixed with 1% formaldehyde. ChIP assays were performed using EZ-Magna-ChIP^TM^ HiSens kit (Millipore). The resulting nuclear extract was sonicated on wet ice and then immunoprecipitated with an appropriate antibody against Gli1(Cell Signaling, 2643S). The following primers set targeting FN1 promoter: forward, 5′-GCATTCTGTAATGGAACTTGTCAG-3′; and reverse, 5′-CAGTTACACACAAAGCAGAGATTT-3′; CK7 promoter: forward, 5′-CTGGCTTCAGCAGCCTGG-3′; and reverse, 5′-GGATGCTTGGAGAGGGAGTG-3′; CK19 promoter: forward, 5′-CCGAGGCTGGAGACAAGT-3′; and reverse, 5′-CCTCTGTTCCAAGCCTGGGT-3′ were designed for quantitative PCR analysis. We utilized 1% of input for all ChIP performed, and to normalize the samples, we employed the following standard equation: 1% input = 2 ^ [(mean Ct input − 6.64) − mean Ct ChIP] * 100.

### Animal Models

All animal procedures were approved by the Mayo Clinic Institutional Animal Care and Use Committee (approval #A3291-01). The EZH2 mice were generated in collaboration with Alexander Tarakhovsky. The EZH2 floxed mouse model was originally generated at the Rockefeller University, following standard homologous recombination techniques to inactivate the endogenous WT locus in embryonic stem cells, generating chimeras, and isolating colony founders carrying the knock-out of this gene. The loxP sites were introduced to flank exons 16–19 which encode the SET domain of EZH2. The genotype was preserved through the archiving of frozen sperm. Frozen sperm was used to recover heterozygous mice in the FVB/NJ strain and subsequently crossed back into a pure C57BL/6 background for more than 20 generations to produce the inbred strain used in this study. Genotyping was confirmed by Southern blot and routinely checked by PCR. The resulting CAG Cre+ EZH2^flox/flox^ mice have global knockout of EZH2 upon tamoxifen exposure. These mice were exposed to tamoxifen for 5 days by intraperitoneal injection and subsequently fed the CDE diet (choline-deficient chow with ethionine-supplemented drinking water) for 3 weeks. Control animals received standard mouse chow and non-supplemented water. Additional control animals included Cag Cre- EZH2^flox/flox^ mice, and mice receiving vehicle in place of tamoxifen (3 animals per group). Under Ketamine/Xylazine anesthesia, mice were sacrificed at week 3 for liver tissue collection or isolation of DRCs.

### Statistical Analysis

Data are presented as the mean ± standard error. Data represents typical experiments reproduced at least three times. Analysis was performed using Graph Stat Prizm software (GraphPad Software, Inc., La Jolla, CA). Statistical analyses used one-way analysis of variance (ANOVA) with a Bonferoni post-test.

## Results

### Pharmacologic and genetic evidence support a role for the Shh pathway in cholangiocyte differentiation

**Fig 2:**
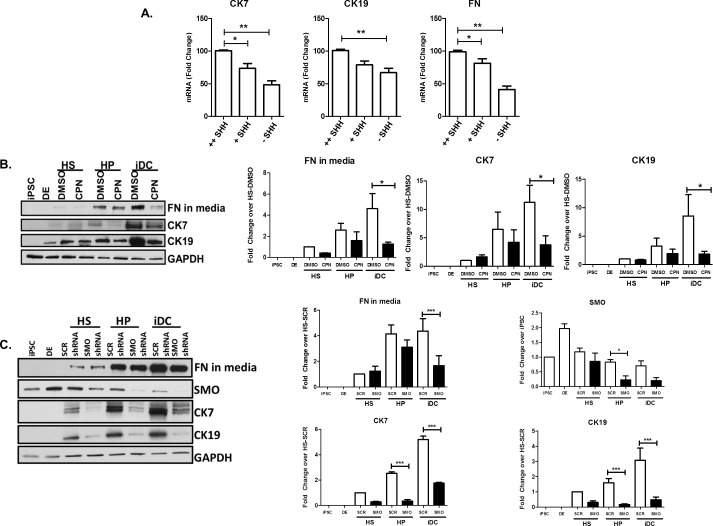
Shh critically mediates cholangiocyte differentiation. A. Reduced Shh dosing reduces mRNA levels of CK7, CK19, and FN (++Shh = 100 ng/ml Shh administered for 8 days, +Shh = 100 ng/ml Shh administered for 4 days, -Shh = no Shh administration). B. Inhibition of Shh signaling using CPN at HS, HP and iDC phases impairs cholangiocyte maturation and FN deposition as assessed by protein levels of CK7, CK19, and released FN. Bar graphs on the right depict the densitometry results showing quantitative reductions in FN, CK7 and CK19. C. SMO shRNA at the HS, HP and iDC phases of differentiation reduces acquisition of CK7 and CK19, and release of FN in media. Bar graphs on the right depict the densitometry results showing quantitative reductions in SMO, FN, CK7 and CK19. The data are shown as means ± standard error. *p < 0.05, ** p <0.01, ***p<0.001.

Using an iPSC-based endodermal cell differentiation model that recapitulates liver maturation (Fig 1A), we find evidence to support a crucial role for Shh in the pathway leading to cholangiocyte-like cells. Briefly, this system is based on the manipulation of iPSC which, in the presence of various morphogens, are allowed to transition through different phases that mimic liver development, namely, DE, HS, HP, and iDC, as previously described [12]. We performed next-generation RNA sequencing at each phase of the iPSC-to-cholangiocyte transition. In the context of this unbiased approach, we noted significant upregulation of Shh (+176.1-fold) as well as a striking induction of FN (+63.2-fold) during biliary specification that correlated with acquisition of the characteristic biliary cytokeratins, CK7 and CK19 (+79.1-fold, 11.0-fold) (Fig 1B). Congruent with this, using quantitative RT-PCR, we confirmed that the expected acquisition of CK7 and CK19 are, indeed, associated with enhancement of both Shh and FN mRNA levels (Fig 1C). These results were also consistent with the finding that reduction or omission of Shh dosing did not allow cells to readily adopt their cholangiocyte-like features, nor to enhance their FN production (Fig 2A). To further determine whether, mechanistically, Shh signaling is necessary for cholangiocyte-like differentiation, we utilized both pharmacologic and genetic approaches. We find that the small drug Shh inhibitor, cyclopamine (5μM), impairs the acquisition of the cholangiocyte markers CK7 and CK19 (CK7: 11.23±3.00 fold-change with DMSO vs 3.76±1.59 with CPN, p<0.05; CK19: 8.56±3. vs 1.87±0.47, p<0.05) as well as FN production (4.64±1.4 vs 1.26±0.18, p<0.05) (Fig 2B). Similar results were obtained upon downregulating the levels of the Shh receptor, Smoothened, via shRNA (SMO: 75% knockdown, CK7: 5.19±0.27 with SCR shRNA vs 1.76±0.07 with SMO shRNA, p<0.05; CK19: 3.1±0.8 vs 0.47±0.18, p<0.05; FN: 4.36±0.97 vs 1.65±0.78, p<0.05) (Fig 2C). Thus, these results suggest that the Shh pathway has the ability to promote cholangiocyte-like cell differentiation and FN secretion using a membrane-to-nucleus mechanism that begins with the activation of canonical cell surface receptors and leads to a transcriptional response.

### Gli1 binding activates transcription of FN, CK7, and CK19

**Fig 3:**
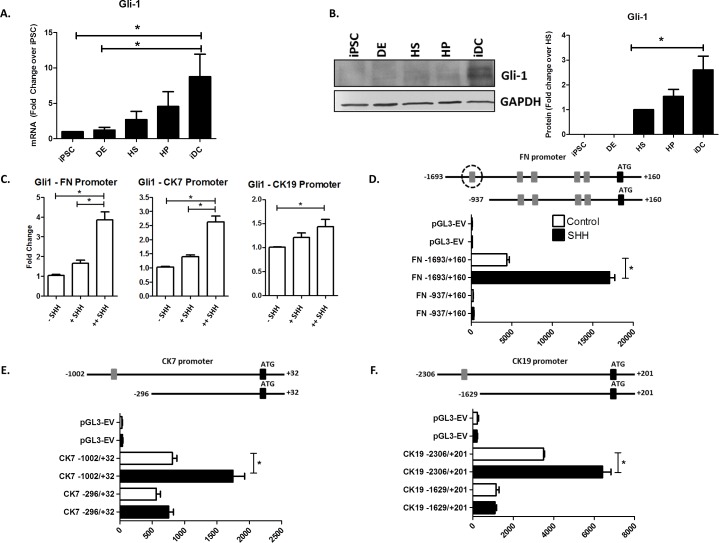
Gli1 binding activates transcription of FN, CK7, and CK19. A. RT-PCR for Gli1 shows increased mRNA during cholangiocyte differentiation. B. Western blotting demonstrates increased Gli1 protein during cholangiocyte differentiation. Bar graphs on the right depict the densitometry results. C. ChIP assay revealing increased Gli1 binding to the FN, CK7, and CK19 promoters with increasing Shh dosing FN (++Shh = 100 ng/ml Shh administered for 8 days, +Shh = 100 ng/ml Shh administered for 4 days, -Shh = no Shh administration). D. Luciferase assays demonstrating basal and Shh-stimulated luciferase activity with the FN promoter before and after truncation. E. Luciferase assays demonstrating basal and Shh-stimulated luciferase activity with the CK7 promoter before and after truncation. F. Luciferase assays demonstrating basal and Shh-stimulated luciferase activity with the CK19 promoter before and after truncation. The data are shown as means ± standard error. *p < 0.05.

Given the robust transcriptional responses to Shh stimulation seen in FN, CK7, and CK19 and to expand our understanding of the mechanisms, we chose to define how the Shh pathway regulates the promoters of these genes. We observed that Shh stimulation in the context of cholangiocyte differentiation promotes an overall increase in Gli1 mRNA (8.8±3.2 fold compared to iPSC) via RT-PCR (Fig 3A) as well Gli1 protein by Western blot (2.7±0.5 fold compared to HS) (Fig 3B). Bioinformatic-assisted mapping of cis-regulatory motifs identified five putative Gli1 binding sites in the FN promoter (GGCC**CCCACC**CACAA), one putative site in the CK7 promoter (GG**CCACCCC**TCT), and another putative site in the CK19 promoter (GG**CCACCCC**TCT). To determine whether and which consensus sites recruit Gli1 in response to Shh, we performed ChIP with and without Shh stimulation. These experiments identified specific functional promoter elements within FN, CK7, and CK19 that bind Gli1. These experiments not only validated the consensus Gli1 sites as functional but also showed significant increases in the binding of this transcription factor in response to Shh treatment (Fig 3C). Subsequent experiments using reporter assays and deletion mutagenesis helped us to characterize the functional impact of the cis-regulatory sites on the regulation of these promoters. Indeed, we find activation of FN promoter activity upon Shh stimulation in the full-length, wild-type construct which is completely abolished upon deletion of the Gli1 binding site (FN full: 4383±535, Shh: 17081±1101, p≤0.05 vs. FN truncation: 212±65, Shh: 307±23, p≤0.05) (Fig 3D). In the CK7 and CK19 promoters, we observe a similar pattern of Shh-based activation that is dependent upon the Gli1 regulatory sites (CK7 full: 810±186.6, Shh: 1749±509 vs CK7 truncation: 562±62, Shh: 754±73 and CK19: 3489±104.6, Shh: 6413±715 vs. CK19 truncation: 1148±250, Shh: 1089±84 p≤0.05) (Fig 3E and 3F). Notably, the degree of activation seen in the luciferase assays is proportional to the observed level of Gli1 binding seen by Chip (FN > CK7 > CK19). Collectively, these experiments demonstrate that these key cholangiocyte gene markers are direct targets of the Shh-Gli1 pathway during iPSC-based biliary differentiation.

### RNA seq identifies EZH2 as a regulator of cholangiocyte differentiation

**Fig 4:**
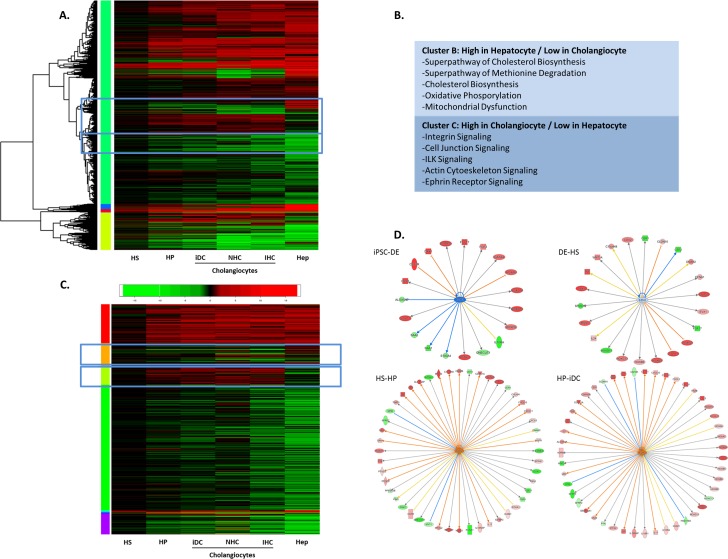
RNA seq identifies EZH2 as a regulator of cholangiocyte differentiation. A. Heatmap of differential expression analysis of RNA sequencing data from HS, HP, iDC, NHC, IHC, and hepatocyte sequencing data. B. Ingenuity pathway analysis of the hepatocyte-specific and cholangiocyte-specific clusters. C. Heatmap of differential expression analysis of epigenetic regulators from RNA sequencing of HS, HP, iDC, NHC, IHC, and hepatocyte sequencing data. D. Analysis of the known EZH2 interactome showing an increasing number of EZH2 target genes upregulated as cholangiocyte differentiation proceeds.

Since transcription factors such as Gli1 do not directly modify the genome, but rather work with key enzymatic complexes to epigenetically regulate gene expression through chromatin remodeling, we next sought to gain insight into complexes that may mediate the function of Shh on cholangiocyte-like differentiation. Previous studies have revealed that in order to activate gene expression, Gli1 works within epigenetic complexes that decorate histone tails with various activating or repressive marks[36]. To expand on this knowledge, we performed differential expression analysis of RNA sequencing data from a variety of sources of cholangiocytes: 1) iDC; 2) the NHC cell line; and 3) isolated human cholangiocytes (IHC). We compared the data from these various cholangiocytes to publicly available hepatocyte sequencing data. The resulting analysis revealed specific clusters of gene expression that could readily distinguish the various cholangiocyte lines from hepatocytes (Fig 4A). Ingenuity pathway analysis of the hepatocyte-specific and cholangiocyte-specific clusters (Clusters B and C, respectively) showed appropriate enrichment for pathways involved in specific hepatocyte or cholangiocyte functions (Fig 4B). This information was then cross-referenced with a proprietary database of known epigenetic regulators (Fig 4C). During cholangiocyte differentiation, we observed changes in negative and positive epigenetic regulators of the K27 residue on histone 3 (e.g. EZH2: -1.2 fold, PCAF: +4.8 fold, P300: +1.9 fold). We subsequently focused on studying the role of EZH2, a key writer of the histone code that enzymatically mediates the H3K27Me3 repressive mark and, in several contexts, antagonizes cell differentiation by acting as an epigenetic barrier[37–39]. We hypothesized, that if functional under the conditions studied here, EZH2 can work as a barrier to cholangiocyte differentiation. Indeed, an analysis of the known EZH2 interactome also demonstrated an increasing number of EZH2 target genes being upregulated as cholangiocyte differentiation proceeds (Fig 4D).

### EZH2 behaves as an epigenetic barrier for cholangiocyte differentiation

**Fig 5:**
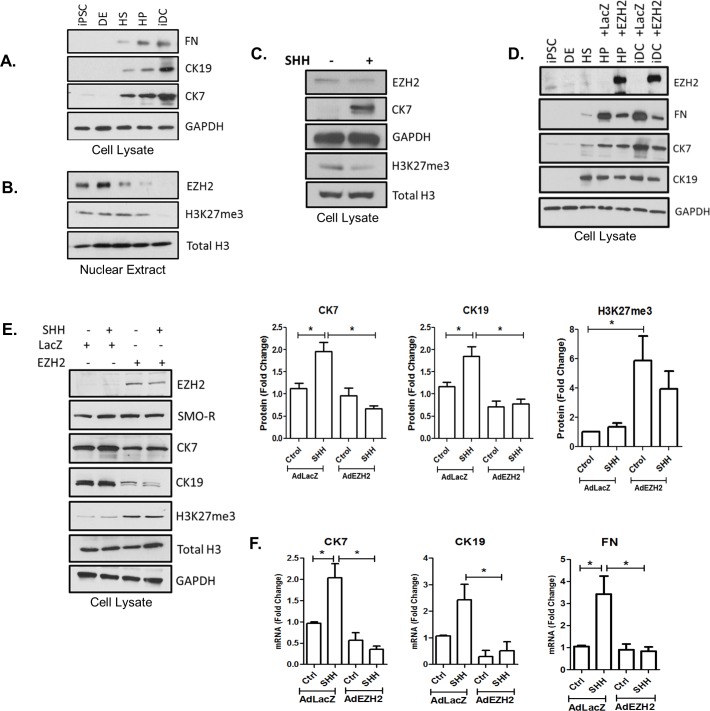
EZH2 behaves as an epigenetic barrier for cholangiocyte differentiation. A. Western blotting on cell lysates demonstrates the acquisition of CK19 and CK7 along with FN production during biliary maturation. B. Western blotting on nuclear extracts demonstrates loss of the EZH2 and the associated H3K27me3 repressive mark as iPSC differentiation proceeds. C. Western blot demonstrates that Shh stimulation of isolated DRCs results in loss of EZH2 and H3K27me3 with concomitant acquisition of CK7. D. Western blotting on cell lysates of iPSC differentiation samples demonstrates that adenoviral overexpression of EZH2 inhibits the acquisition of CK7 and CK19 as well as FN secretion. E. Western blotting of isolated ductular reactive cells shows that EZH2 overexpression correlates with H3K27me3 and loss of CK7 and CK19. Bar graphs on the right side represent densitometry of CK7, CK19, and H3K27me3 protein levels. F. Quantitative RT-PCR shows that EZH2 overexpression correlates with loss of CK7, CK19, and FN mRNA expression. The data are shown as means ± standard error. *p < 0.05.

**Fig 6:**
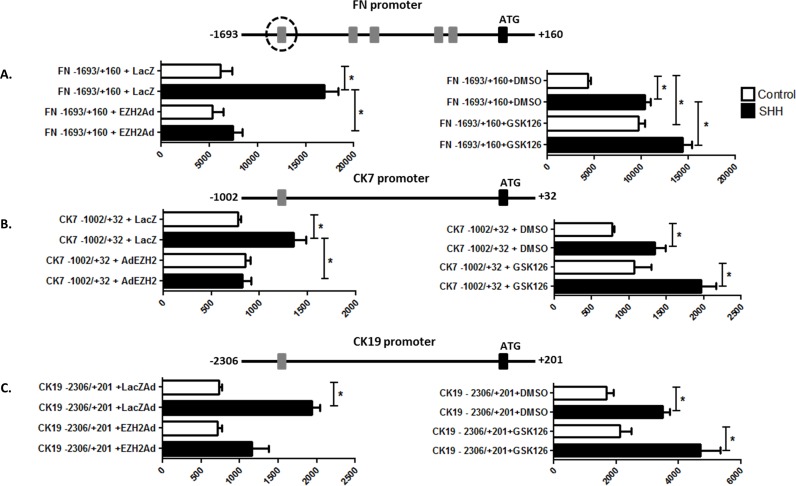
EZH2 inactivates transcription of FN, CK7, and CK19. A. C. Luciferase assays demonstrating basal and Shh-stimulated luciferase activity with the FN, CK7, and CK19 promoters with or without EZH2 overexpression. B. Luciferase assays demonstrating basal and Shh-stimulated luciferase activity with the FN, CK7, and CK19 promoters in the presence and absence of pharmacologic EZH2 inhibition. The data are shown as means ± standard error. *p < 0.05.

We next evaluated the direct effects of EZH2 on cholangiocyte differentiation using both the iPSC system and isolated DRCs. We find that cholangiocyte differentiation in the iPSC system, including the acquisition of CK7, CK19, and FN (Fig 5A), is associated with a reduction of EZH2 in nuclear extracts (Fig 5B). Notably, this change starts to become apparent at the HS and HP phases when Shh stimulation is present. Congruently, this phenomenon is also accompanied by a decrease in the repressive H3K27me3 mark (Fig 5B). Similar results were obtained in isolated DRCs treated with exogenous Shh (Fig 5C), suggesting that Shh activation abrogates EZH2-mediated transcriptional repression thereby contributing to the activation of cholangiocyte gene markers. This idea was further supported by the finding that adenoviral-induced upregulation of EZH2 during iPSC differentiation significantly impairs the acquisition of these cholangiocyte markers (Fig 5D). Furthermore, in isolated DRCs, EZH2 prevented the Shh-mediated activation of cholangiocyte differentiation at the protein (Fig 5F—CK7: LacZ: 1.9±0.27 vs. AdEZH2: 0.58±0.13, CK19: LacZ: 1.8±0.43 vs. AdEZH2: 0.79±0.1 and H3K27me3: LacZ: 1.83±0.50 vs. AdEZH2: 3.8±0.97) (Fig 5E) and mRNA levels (FN: LacZ: 3.4±0.9 vs. AdEZH2: 0.84±0.2, CK7: LacZ: 2.0±0.3 vs. AdEZH2: 0.35±0.1 and CK19: LacZ: 2.4±0.6 vs. AdEZH2: 0.5±0.3, p≤0.05). We also observed that upon adenoviral overexpression of EZH2, the Shh-induced luciferase activity driven by the FN, CK7, and CK19 promoter constructs was abolished (Fig 6A: FN—AdLacZ: 16917±1448 vs. AdEZH2: 5295±1197, CK7- AdLacZ: 1354±127 vs. AdEZH2: 820±91, CK19- AdLacZ: 1938±102 vs. AdEZH2: 1157±380, p≤0.05), suggesting that the constructs are adequately chomatinized in this *in vitro* system. In contrast, pharmacologic inhibition of EZH2 with GSK126 enhanced the Shh-induced luciferase activity (Fig 6B: FN- DMSO: 10373±1652 vs. GSK126: 14416±2538, CK7—DMSO: 1350±349 vs. GSK126: 1965±450 and CK19- DMSO: 3500±4541 vs. GSK126: 4701±637, p≤0.05). Collectively, this data, for the first time, identifies EZH2 as potential chromatin-based barrier to the activation of transcriptional events that are necessary for the acquisition of cholangiocyte-like properties.

### Genetic inactivation of EZH2 increases biliary expansion in vivo

**Fig 7:**
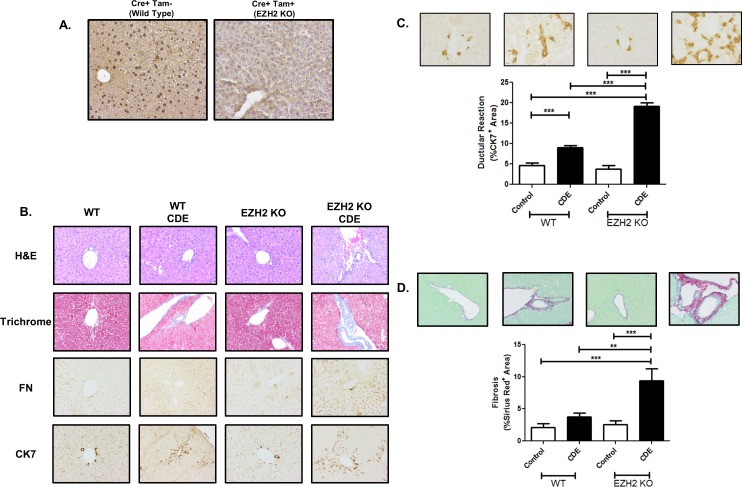
EZH2 KO exacerbates CDE-induced biliary expansion. A. Immunohistochemistry for EZH2 demonstrates robust nuclear staining for EZH2 in the liver of wild type animals that is absent in the knockout animals. B. H&E staining, trichrome staining, and immunohistochemistry demonstrated enhanced ductular reaction following CDE feeding in EZH2 KO animals including peri-portal fibrosis, ductular proliferation, fibronectin deposition, and expansion of CK7 positive DRCs. C. Quantification of the CK7+ area shows increased CDE-induced ductular reaction following EZH2 KO. D. Quantification of the Pico Sirius Red+ area shows increased CDE-induced peri-portal fibrosis following EZH2 KO. The data are shown as means ± standard error. ** *p* < 0.01, *** *p* < 0.001.

To define the role of EZH2 as an important regulator of cholangiocyte biology *in vivo*, we utilized the CDE diet to promote liver injury and ductular reaction. We utilized a tamoxifen-inducible EZH2 knock out animal, in which expression of an inducible Cre-recombinase can be activated in the live animals during CDE feeding, resulting in global knockout of EZH2. Immunohistochemistry experiments confirmed efficient loss of EZH2 nuclear staining in the liver of the KO animals (Fig 7A). More importantly, we find that following CDE feeding, knockout animals show an exaggerated phenotype including enhanced ductular reaction, including biliary fibrosis, FN deposition, and more robust CK7-positive biliary expansion (Fig 7B). As expected, Cre negative control animals lack this exaggerated response (S1 Fig). High power trichrome stained images confirm ductular proliferation encased in a dense ECM in the CDE-fed knockout animals (S2 Fig). In order to more precisely define the observed changes, we quantified the amount of ductular reaction and fibrosis, by measuring the CK7+ and Sirius Red+ area, respectively (Fig 7C and 7D). The results showed a 2-fold increase in CDE-induced ductular proliferation in the KO animals compared to the WT controls (WT CDE: 8.98 ± 1.49, KO CDE: 19.09 ± 2.68) along with a 2.5-fold increase in CDE-induced peri-portal fibrosis in the KO animals compared to the WT controls (WT CDE: 3.79 ± 0.57, KO CDE: 9.38 ± 1.89). These *in vivo* experiments are congruent with our cellular and transcriptional studies and together demonstrate a barrier function of EZH2, which when lifted, supports cholangiocyte maturation and leads to an expansion of the ductular compartment of the murine liver.

## Discussion

**Fig 8:**
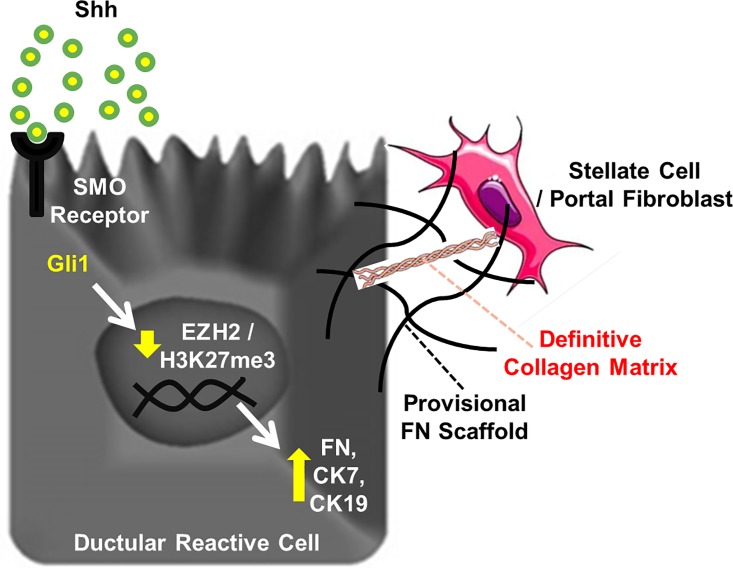
Working Model. An epigenetic program, initiated by Shh, and mediated by EZH2 loss, links cholangiocyte maturation to the deposition of a provisional FN matrix, shedding light on the simultaneous processes of biliary repair and peri-portal scar formation. FN may serve as a provisional scaffold for subsequent recruitment and activation of hepatic stellate cells or portal myofibroblasts.

In recent years, we have seen significant growth in the field of biliary regenerative medicine, including a new-found ability to reproduce the complex sequence of events by which cholangiocytes are formed from stem cells [11–14]. However, it is critically important that we dissect, in detailed mechanistic depth, the signaling pathways and epigenetic events that coordinate the stem cell-to-cholangiocyte transition. These conceptual advances will improve our understanding of biliary development, tissue homeostasis in adult liver, and biliary repair mechanisms and will allow the optimization of future regenerative medicine protocols. In this context, the current study provides several novel conceptual advances: 1) Shh represents a key morphogen that regulates expansion of the biliary compartment; 2) the Shh-Smo-Gli1 signaling axis overcomes an EZH2-dependent epigenetic barrier and initiates cholangiocyte maturation and ECM deposition; 3) Gli1 binding to specific and previously unknown sites within the FN, CK7, and CK19 promoters antagonizes an EZH2-dependent repressive complex and activates transcription; and 4) EZH2 KO exacerbates the phenotype of CDE-fed mice with in terms of ductular reaction and peri-portal fibrosis. Overall, these findings define an epigenetic mechanism, initiated by Shh, and mediated by EZH2, that drives biliary expansion and sheds additional light on the simultaneous processes of biliary maturation and peri-portal scar formation (Fig 8).

Derivation of cholangiocytes from pluripotent stem cells is an emerging and evolving technology. While it is remarkable that disparate differentiation protocols can lead to mature cholangiocytes [11–14], it is likely that additional fine-tuning of the various protocols will be required in order to optimize the cholangiocyte phenotypes and more precisely recapitulate the natural progression from precursor cell types. Our study outlines an important role for Shh signaling in this process. Indeed, early studies of hepatic differentiation have shown that inclusion of Shh tended to result in the emergence of biliary elements in the differentiated progeny [40]. We also note that inclusion of Shh in our differentiation protocol precluded efficient expression of hepatocyte markers [12]. Furthermore, repetitive dosing of Shh further promotes the acquisition of cholangiocyte features. In line with this, others have demonstrated that Shh inhibits hepatocyte differentiation in fetal liver progenitors [16]. In aggregate, these observations suggest a key role for the Shh pathway in the fate decision of biliary precursors. While other published protocols [11, 13, 14] describing cholangiocyte differentiation from iPSCs do not exogenously activate the Shh pathway, it was not reported whether or not this pathway is indirectly activated in these systems and this remains to be determined. Our work here confirms Shh as one key signaling pathway that can promote biliary features in multiple cell systems while mechanistically extending our understanding of the transition, linking Shh activation to an EZH2-mediated epigenetic regulatory program.

The PRC2 complex is a large, multi-subunit, epigenetic regulatory complex that silences gene expression across a diverse array of processes including embryologic development, cellular differentiation, pluripotency, and neoplasia. PRC2 enzymatically mediates the tri-methylation of H3K27 via the histone methyltransferase, EZH2, a key writer of the histone code. This event recruits PRC1 and represses gene expression through chromatin compaction and other mechanisms [41]. We initially noted that EZH2 levels were suppressed as differentiation proceeded from iPSC to progenitor phases and was further reduced in mature cholangiocytes. This is in line with current concepts of EZH2 as a marker of “stemness” and indeed it is thought to be associated with de-differentiation in many malignancies [42], including cholangiocarcinoma (CCA) [43], and is therefore, a common target of cancer therapy research. Our study also delineates, for the first time, a mechanism whereby Shh signaling can de-activate the PRC by Gli1-based antagonism of EZH2 at the promoter binding level. These findings could potentially have implications in other biological processes, particularly biliary neoplasia. It is, however, important to note that recent studies also define a non-canonical Shh pathway in CCA that does not involve Gli1 and actually promotes the aggressiveness of CCA through effects on chemotaxis [23].

It is also noteworthy that this report is the first to identify an epigenetic mechanism for Shh-mediated transcription of FN, CK7, and CK19. While a few scattered reports have incidentally noted that Shh may increase matrix proteins in the contexts of pulmonary and renal fibrosis [44, 45], we now directly demonstrate functional Gli1 binding sites in the FN, CK7, and CK19 promoters that are capable of activating transcription. Furthermore, we identify that Shh can activate Gli1-mediated disruption of an epigenetic silencing complex containing EZH2 in these promoters to drive context-specific gene expression patterns. While liver fibrogenesis is mediated primarily by collagen deposition by hepatic stellate cells and various other myofibroblasts [46], FN is thought to recruit these cells and serve as a preliminary scaffold onto which collagen fibrils can subsequently assemble in the development of hepatic fibrosis (Fig 8). Indeed, recent studies show that inhibition of FN can actually attenuate liver fibrosis [47]. Our work is consistent with this and shows that animals lacking EZH2 have an exaggerated DRC response to the CDE diet that also promotes peri-portal fibrosis. This is especially intriguing since the work identifies several new molecular targets upstream of FN that may provide opportunities to intervene prior to stellate cell activation and collagen deposition, at a stage when biliary fibrosis may be more reversible.

## Supporting Information

S1 Table Primary Antibodies. (DOCX)

S2 Table RT-PCR Primers. (DOCX)

S1 Fig Cre Negative Control Animals. H&E staining, trichrome staining, and immunohistochemistry in Cre negative control animals.
(DOCX)

S2 Fig High Power Trichrome Images. 40X images of Masson’s Trichrome staining. ^PV^portal vein, *bile duct.
(DOCX)

## Abbreviations

ANOVA: analysis of variance
ChIP: chromatin Immunoprecipitation
CDE: choline-deficient ethanolamine-supplemented
CAG Cre: Cre recombinase driven by the chicken beta actin gene
CK: cytokeratin
CPN: cyclopamine
DE: definitive endoderm
DRCs: ductular reactive cells
EPCAM: epithelial cell adhesion molecule
EZH2: enhancer of zeste homolog 2
EZH2Ad: EZH2 adenovirus
FN: fibronectin
HS: hepatic specification
HP: hepatic progenitor
iPSC: induced pluripotent stem cells
iDC: induced pluripotent stem cell-derived cholangiocytes
IHC: isolated human cholangiocytes
NHC: normal human cholangiocytes
PRC2: polycomb repressive complex 2
SMO-R: smoothened receptor
Shh: sonic hedgehog
H3K27me3: tri-methylation of lysine 27 on histone 3
