# Supplementary material for: Hedgehog Signaling Overcomes an EZH2-Dependent Epigenetic Barrier to Promote Cholangiocyte Expansion
Source: PLoS One. 2016 Dec 9;11(12):e0168266. doi: 10.1371/journal.pone.0168266 (PMC5148157; doi:10.1371/journal.pone.0168266)
Supplement: S1 Table — (DOCX) [file pone.0168266.s001.docx]

**S1 Table**: Primary Antibodies

| **Target** | **Antibody Company** | **Cat No.** | **Working Dilution (Western)** | **Working Dilution (IHC)** | **Molecular weight (Kd)** |
| --- | --- | --- | --- | --- | --- |
| CK7 | Santa Cruz Biotechnology | sc-53263 | 1:250 | 1:100 | 51 |
| CK19 | Sigma Aldrich | SAB 1400148 | 1:1000 |  | 44 |
| EZh2 | Millipore | 17-662 | 1:1000 | 1:250 | 100 |
| FN | Santa Cruz Biotechnology | sc-9068 | 1:1000 |  | 220 |
| FN | BD Biosciences |  |  | 1:250 |  |
| Shh | Cell Signaling | 2207S | 1:500 |  | 45(19) |
| Smo-R | Abcam | ab72130 | 1:1000 |  | 85 |
| Total H3 | Abcam | ab1791 | 1:1000 |  | 15 |
| H3K27 | Abcam | ab6002 | 1:1000 |  | 15 |
| LGR5 | Millipore | ABD17 | 1:1000 |  | 95 |
| EpCaM | Santa Cruz Biotechnology | sc-53532 |  |  | 35 |
| Gli-1 | Cell Signaling | 2643S | 1:500 |  | 118 |
| Cd63 | Santa Cruz Biotechnology | sc-15363 | 1:200 |  | 26 |
| Cd81 | Santa Cruz Biotechnology | sc-9158 | 1:200 |  | 26 |
| TSG101 | Santa Cruz Biotechnology | sc-7964 | 1:200 |  | 43 |
| TfR | Invitrogen | 13-6800 | 1:1000 |  | 95 |
