# Supplementary material for: Hedgehog Signaling Overcomes an EZH2-Dependent Epigenetic Barrier to Promote Cholangiocyte Expansion
Source: PLoS One. 2016 Dec 9;11(12):e0168266. doi: 10.1371/journal.pone.0168266 (PMC5148157; doi:10.1371/journal.pone.0168266)
Supplement: S2 Table — (DOCX) [file pone.0168266.s002.docx]

**S2 Table**: RT-PCR Primers

| **Human Target Gene** |  | **Sequence** |
| --- | --- | --- |
| CK19 | Sense  Antisense | GCGAGCTAGAGGTGAAGATC  AATCCTGGAGTTCTCAATGGTG |
| CK7 | Sense  Antisense | CTTCTGTCTTACGGCGACTAAG  AGGTCTAACTCTACTGTCAGGG |
| FN | Sense  Antisense | GATAAATCAACAGTGGGAGC  CCCAGATCATGGAGTCTTTA |
| EZH2 | Sense  Antisense | CCACAGTGTTACCAGCATTTG  ACTGTTATTGGGAAGCCGTC |
| Shh | Sense  Antisense | CTACGAGTCCAAGGCACATATC  CAGGTCCTTCACCAGCTTG |
| Gli-1 | Sense  Antisense | CCAACTCCACAGGCATACAG  ATACACAGATTCAGGCTCACG |

| **Mouse Target Gene** |  | **Sequence** |
| --- | --- | --- |
| CK19 | Sense  Antisense | CTCCCGAGATTACAACCACTAC  GTTCTGTCTCAAACTTGGTTCTG |
| CK7 | Sense  Antisense | TGAGATTGCGGAGATGAACC  CGATGCTGGACTCTAACTTGG |
| FN | Sense  Antisense | ACCTCGACAAGTGCATGG  CCAAAACCAGGACCAGACTAAG |
| EZH2 | Sense  Antisense | TCCCGTTAAAGACCCTGAATG  TGAAAGTGCCTACCTGATCC |
