# Supplementary material for: Hedgehog Signaling Overcomes an EZH2-Dependent Epigenetic Barrier to Promote Cholangiocyte Expansion
Source: PLoS One. 2016 Dec 9;11(12):e0168266. doi: 10.1371/journal.pone.0168266 (PMC5148157; doi:10.1371/journal.pone.0168266)
Supplement: S1 Fig — H&E staining, trichrome staining, and immunohistochemistry in Cre negative control animals. (DOCX) [file pone.0168266.s003.docx]

**S1 Fig:** Cre Negative Control Animals.
