# Supplementary figures and images for: Hedgehog Signaling Overcomes an EZH2-Dependent Epigenetic Barrier to Promote Cholangiocyte Expansion
Source: PLoS One. 2016 Dec 9;11(12):e0168266. doi: 10.1371/journal.pone.0168266 (PMC5148157; doi:10.1371/journal.pone.0168266)

**S2 Fig:** High Power Trichrome Images.


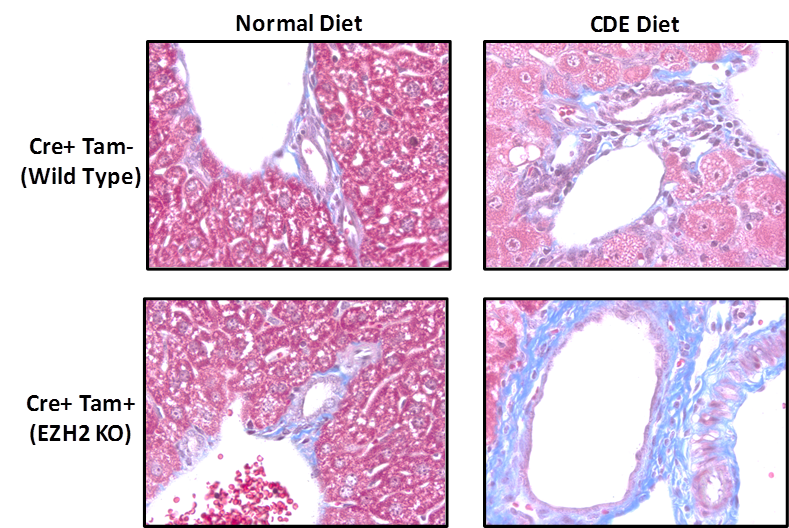


PV

PV

PV

PV

*

*

*

*

Supplement: S2 Fig — 40X images of Masson’s Trichrome staining. PVportal vein, *bile duct. (DOCX) [file pone.0168266.s004.docx]
